# Ag/AgCl/MIL-101(Fe) Catalyzed Degradation of Methylene Blue under Visible Light Irradation

**DOI:** 10.3390/ma12091453

**Published:** 2019-05-05

**Authors:** Yun Liu, Yuanhong Xie, Mingjin Dai, Qingjiao Gong, Zhi Dang

**Affiliations:** 1 Department of Environmental Science and Engineering, College of Environment and Resources, Xiangtan University, Xiangtan 411105, China; xieyuanhong0222@126.com (Y.X.); dmj19930611@163.com (M.D.); qingjiaogong@163.com (Q.G.); 2 School of Environment and Energy, South China University of Technology, Guangzhou 510006, China; chzdang@scut.edu.cn

**Keywords:** photo-Fenton, Ag/AgCl/MIL-101(Fe), Box–Behnken design, methylene blue

## Abstract

A novel photo-Fenton catalyst named Ag/AgCl/MIL-101(Fe) was synthesized by the method of precipitation and photo reduction and characterized by X-ray diffraction patterns (XRD), Brunauer-Emmett-Teller (BET) measurements, Fourier transform infrared spectra (FTIR), scanning electron microscopy with energy dispersive X-ray spectroscopy (SEM-EDX), X-ray photoelectron spectroscopy (XPS) and UV-vis diffuse reflectance spectra. Moreover, the catalytic activity of the synthesized catalyst was tested using methylene blue (MB) as the target pollutant. The obtained results illustrated that the plasmonic material Ag/AgCl was successfully loaded on MIL-101(Fe) and the obtained catalyst exhibited an excellent catalytic activity under visible light at the neutral pH. According to the analyses of Plackett-Burman and Box-Behnken design, the optimum conditions for MB degradation were obtained. Under these conditions, the MB decolorization and mineralization efficiencies could reach to 99.75% and 65.43%, respectively. The recycling experiments also showed that the as-prepared catalyst displayed good reusability. In addition, the possible reaction mechanisms for the heterogeneous photo-Fenton system catalyzed by Ag/AgCl/MIL-101(Fe) were derived. The synthesized catalyst provides a promising approach to degrade organic pollutants in waste water.

## 1. Introduction

Dyes are widely used in a number of industries, such as printing, painting, textile, paper, leather, etc. [1]. It is estimated that commercial dyes about 7 × 10^5^ t are produced worldwide each year and about 5–10% of them are eventually lost in the waste water [2]. This waste water, if not properly treated, could result in considerable damage to aquatic life and human health. Most of the dyes are hardly removed from effluents by conventional biological (activated sludge) processes [3]. Thus, an efficient method of treating dye wastes is urgently required.

Heterogeneous photo-Fenton process is considered to be an attractive method for dyes treatment because of its generation of powerful active radicals, a relatively wide range of applications and the easy separation of the catalysts from a water stream. In the past few decades, various materials including FeOOH [4], Fe_2_O_3_ [5] and supported iron catalysts [6,7], have been used as heterogeneous photo-Fenton catalysts to degrade recalcitrant compounds. These catalysts, however, suffer from poor H_2_O_2_ utilization and low catalytic activity due to their limited exposed active sites [8]. Therefore, the development of highly effective heterogeneous photo-Fenton catalysts with large numbers of exposed active sites has emerged as an urgent task in this field.

Metal–organic frameworks (MOFs), a kind of porous material with high surface area, have been extensively studied in catalysis [9], gas storage [10], adsorption [11] and separation [12]. MOFs, also called porous coordination polymers, are built from organic linkers and metal ions/clusters, which belong to an extraordinary family of crystalline porous materials. The detailed model structure of MOFs can be seen in the previous reference [13]. Researchers have shown that MOFs containing iron species can be used as heterogeneous Fenton catalysts due to their attractive property of wide distribution of single iron sites and wide pH range application [14]. However, the catalytic efficiency of pure MOFs still needs to be improved since these materials contain only Fe(III) species with weak Fenton activity [15]. That is why supplementary UV-light needs to be applied for increasing the reduction of Fe(III) to Fe(II) and thus enhancing the Fenton catalytic efficiency of MOFs. However, the UV-light accounts for only 3–5% of the incoming solar energy, while large quantities of electrical power are required to use artificial UV-light sources, the widely expected practical applications of iron–based MOFs as photo-Fenton catalysts have been restricted [16]. As such, the enhancement of the photo-Fenton catalytic efficiency of iron–based MOFs under visible light irradiation is very meaningful for their application.

Coupling with these iron–based MOFs and plasmonic materials might be an effective strategy to solve this problem. Plasmonic materials are kinds of materials with surface plasmon resonance (SPR) effects [17]. When light interacts with these materials, the cloud of free electrons in these materials can support a wave of charge density fluctuations on the surface of these materials [18]. This phenomenon is called a surface plasmon wave, which makes plasmonic materials able to absorb strongly visible light and leads to greatly enhance light-matter interactions. As we know, plasmonic photocatalysts, e.g., Ag/AgX (X = Cl,Br,I), have received widespread interest owing to their high photocatalytic ability under visible light conditions as a result of their surface plasmon resonance [19]. Nevertheless, Ag/AgX composites suffer from the quick recombination of the electron and hole pairs, resulting in the losses of photocatalytic efficiency [20]. On the other hand, recent studies showed Fe(III) can act as electron acceptor to lower the recombination of photo induced electron–hole pairs produced by semiconductors [21]. Inspired by the above studies, we attempt to modify MIL-101(Fe) with Ag/AgCl, which may accelerate the reduction of Fe(III) to Fe(II) by photo-induced electrons and then improve the visible light Fenton catalytic activity of MIL-101 (Fe), which is a kind of iron-based MOFs formed by the coordination of ferric ions and terephthalic acid [22].

In this work, MIL-101(Fe) was used as a host material to load Ag/AgCl to synthesize a new catalyst called Ag/AgCl/MIL-101(Fe) with high visible light driven-Fenton catalytic activity under neutral pH. Methylene blue (MB) was used as a target contaminant to investigate the photo-Fenton catalytic activity of Ag/AgCl/MIL-101(Fe). Box–Behnken design (BBD) method was employed to optimize the effects of variables such as initial MB concentration, H_2_O_2_ concentration, irradiation intensity and catalyst dosage on the degradation of MB. Additionally, the possible reaction mechanisms involved in the photo-Fenton system were discussed.

## 2. Experimental

### 2.1. Materials and Reagents

The MB was obtained from Kelong Chemical Reagents Company (Chengdu, China). Silver nitrate (AgNO_3_), FeCl_3_·6H_2_O, terephthalic acid (H_2_BDC), dimethylformamide (DMF), absolute ethanol (CH_3_CH_2_OH), sodium chloride (NaCl), HNO_3_ and NaOH used in the experiments were of commercially available analytical grade.

### 2.2. Catalyst Preparation

#### 2.2.1. The preparation of MIL-101 (Fe)

MIL-101 (Fe) was prepared by the method of hydrothermal synthesis [23]: 1.714 g of H_2_BDC, 0.824 g of FeCl_3_·6H_2_O and 15 mL of DMF were placed in a 60 mL Teflon-lined stainless steel autoclave and maintained at 110 °C for 20 h. After cooling down to ambient temperature, the suspension was filtered. The resulting yellow powder was rinsed with ethanol and deionized water repeatedly, and then vacuum dried overnight at 60 ℃ to obtain MIL-101 (Fe).

#### 2.2.2. The preparation of Ag/AgCl/MIL-101 (Fe)

The Ag/AgCl/MIL-101 (Fe) catalyst was synthesized according to the precipitation - photoreduction method [24]. Firstly, 1.141 g of AgNO_3_ and 2.412 g of NaCl was respectively dissolved in 100 mL water to prepare 0.3 mol/L AgNO_3_ solution and 0.2 mol/L NaCl solution. Secondly, 1.0 g of MIL-101(Fe) was dispersed in 500 mL of AgNO_3_ solution with stirring at ambient temperature for 30 min. Then, 25 mL of NaCl solution was dropped to the mixture solution and further stirred for 30 min. Subsequently, this mixture was illuminated with 500 W Xe lamp for 60 min so as to reduce part of Ag^+^ into Ag^0^. Finally, the product was filtrated, washed, and dried overnight at 60 °C. For comparison, the plasmonic photocatalyst of Ag/AgCl was also prepared by the same method without the addition of MIL-101(Fe).

### 2.3. Characterization

X-ray diffraction patterns (XRD) of the catalysts were recorded by a diffractometer (RigakuD/max-2550 VK/PC) equipped with a Cu Kα source. The N_2_ adsorption/desorption isotherms were obtained on a Micromeritics Tristar 3020 instrument. The specific surface areas were analyzed by the Brunauer-Emmett-Teller (BET) method. The Fourier transform infrared spectra (FTIR) were performed with a FTIR spectrometer (Nicolet 380, Thermo Nicolet, Madison, WI, USA) in KBr medium in the region of 400–4000 cm^−1^. The morphology and chemical composition of the samples were investigated by using a Hitachi S-530 scanning electronic microscope (Hitachi Scientific Instruments, Tokyo, Japan) with an OXFORD Link-ISIS-300 energy dispersive X-ray spectrometer (SEM-EDX, Oxford Instruments, Oxford, UK). The chemical states of the samples were analyzed by an ESCALAB 250 X-ray photoelectron spectroscopy (XPS, Thermo-VG Scientific, West Sussex, UK) with an Al Kα X-ray source. UV-vis diffuse reflectance spectra were acquired with a Shimadzu UV-2500 spectrophotometer (Shimadzu Co., Kyoto, Japan) using BaSO_4_ as the reference material. Electron spin resonance (ESR) spectra were recorded on a JES-FAESR spectrometer (JEOL Ltd., Tokyo, Japan), using 5,5-dimethyl-1-pyrroline N-oxide (DMPO) as the spin trapping reagent. The photocurrent responses were measured on an electrochemical workstation (CHI660, CH Instruments, Austin, TX, USA) with a three-electrode system, utilizing a Pt electrode as the counter electrode, and a saturated calomel electrode as the reference electrode, the working electrode was a glassy carbon electrode modified by the catalyst to be measured.

### 2.4. Photo-Fenton Catalytic Experiments and Analytical Methods

The photo-Fenton catalytic experiments were performed in a photochemical reaction instrument, in which a 500 W Xenon lamp with a 420 nm cut-off glass filter was employed as the visible light source. In these experiments, 10 mg of photo-Fenton catalyst was suspended into 100 mL MB solution and stirred for 30 min in the dark condition to establish an adsorption/desorption equilibrium. The reaction was started when H_2_O_2_ was added to the reaction solution and the Xenon lamp was turned on. At the given time intervals, the analytical samples were extracted and centrifuged (10,000 rpm, 5 min) to remove the precipitate. The MB concentration left in the supernatant was determined with a 722S spectrophotometer (Shanghai Youke Instrument Co, Ltd., China) at its λ_max_ = 664 nm. The total organic carbon (TOC) of the aqueous solution was determined by a Shimadzu TOC-L CPH TOC analyzer (The decolorization efficiency and TOC removal efficiency of MB were calculated as follows (Equations (1) and (2)):

(1) $$\mathrm{Decolorization}\;\mathrm{efficiency}\left(\%\right)=\frac{C_{0}-C_{t}}{C_{0}}\times 100$$

(2) $$\mathrm{TOC}\;\mathrm{removal}\;\mathrm{efficiency}\left(\%\right)=\frac{{\mathrm{TOC}}_{0}-{\mathrm{TOC}}_{t}}{{\mathrm{TOC}}_{0}}$$

where C_0_ and TOC_0_ are the dye concentration and the TOC concentration at the time of adsorption–desorption equilibrium, respectively. C_t_ and TOC_t_ are the dye concentration and the TOC concentration at certain time t during the photo-Fenton reaction process, respectively.

Plackett-Burman (P-B) design is useful for screening key factors from a multivariable system rapidly [25]. In the present study, the influence of eleven variables including six actual variables and five dummy variables on MB degradation in the photo-Fenton process were investigated using P-B design. Each independent variable was evaluated at two levels, a high (+) and a low (−) level, which are shown in Table S1 in the Supplementary Material. The range of values were selected according to the preliminary experiments. The effects of the variables on the MB degradation are listed in Table S2 in the Supplementary Material. A detailed analysis of the regression coefficients showed that initial dye concentration, hydrogen peroxide concentration, irradiation intensity and catalyst dosage had significant effects on MB degradation in the photo-Fenton process. It is worth noting that the changing pH from 5–9 has insignificant effect on the MB degradation, indicating the synthesized catalyst is stable over a relatively wide pH range near neutrality.

The optimal experimental conditions for MB degradation were further studied by using the Box–Behnken design (BBD), which was established using Design Expert Software (version 7.0). Based on the above P-B design, four critical parameters of initial dye concentration, hydrogen peroxide concentration, irradiation intensity and catalyst dosage were coded with low (−), middle (0), and high (+) levels in BBD (shown in Table 1), while the responses were expressed as % of MB decolorization efficiency and TOC removal efficiency after 2 h reaction. In all of these systems, the initial pH was set at 7.

The BBD results were fitted with the following quadratic model (Equation (3)):

(3) $$Y=k_{0}+k_{a}A+k_{b}B+k_{c}C+k_{\mathrm{ab}}\mathrm{AB}+k_{\mathrm{ac}}\mathrm{AC}+k_{\mathrm{bc}}\mathrm{BC}+k_{\mathrm{aa}}A^{2}+k_{\mathrm{bb}}B^{2}+k_{\mathrm{cc}}C^{2}$$

The significance of each parameter has been illustrated by our previous report [26].

## 3. Results and Discussion

### 3.1. Characterization of the Catalysts

**Figure 1:**
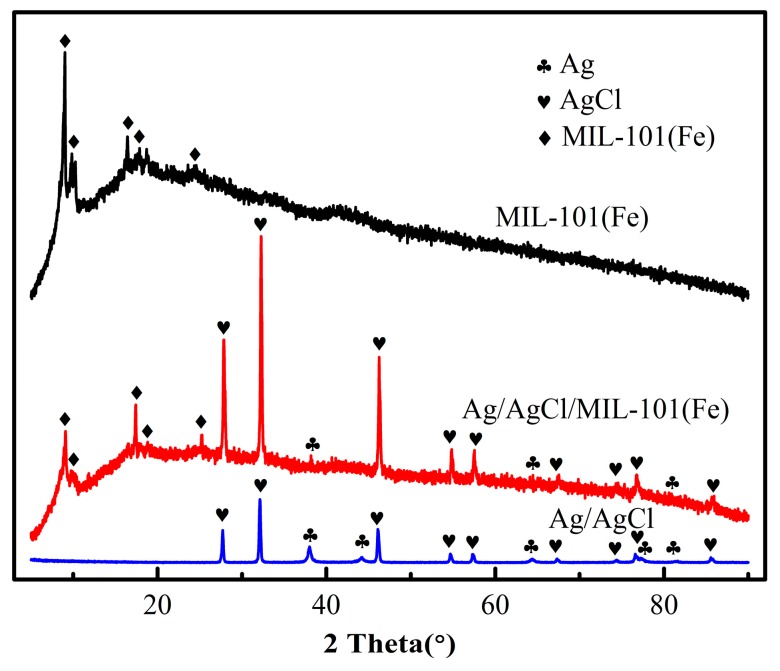
X-ray diffraction patterns of the Ag/AgCl, MIL-101(Fe) and Ag/AgCl/MIL-101(Fe).

The crystallographic structures of the as-prepared Ag/AgCl, MIL-101(Fe) and Ag/AgCl/MIL-101(Fe) sample were determined by powder XRD (Figure 1). The collected diffraction pattern of MIL-101(Fe) at 2θ = 8.95°, 9.84°, 16.43°, 18.79° and 24.65° were matched well with the reported pattern [27], indicating that MIL-101(Fe) had been successfully prepared. Beside the characteristic signal of MIL-101 at 2θ = 1–25°, some new diffraction peaks appear at 2θ = 27.89°, 32.14°, 46.38°, 54.74°, 57.52°, 67.52°, 76.76° and 85.84° in the XRD pattern of Ag/AgCl/MIL-101(Fe), which respectively correspond to the (111), (200), (220), (311), (222), (400), (331), (420) and (422) crystal facets of AgCl [28]. Moreover, the peak at 38.17°, 64.45° and 81.54° can be assigned to the (111), (220) and (222) crystal facets of Ag [29]. All of the above peaks corresponding to Ag and AgCl can be found in the XRD pattern of Ag/AgCl. The XRD results prove the successful fabrication of Ag/AgCl/MIL-101(Fe) composite.

**Figure 2:**
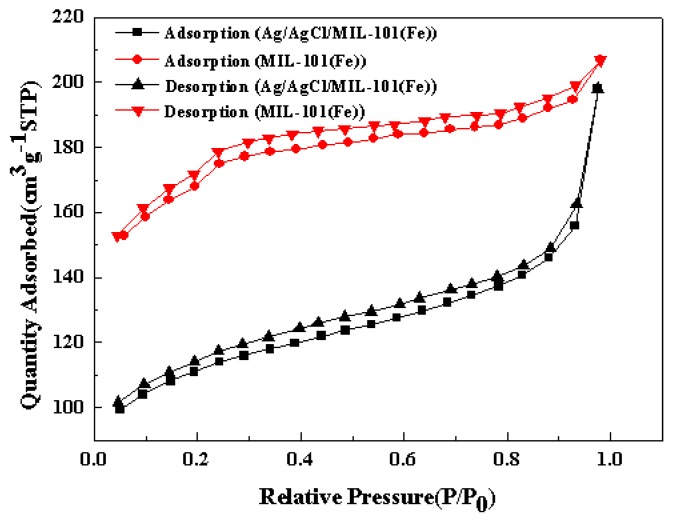
Nitrogen adsorption-desorption isotherms of MIL-101(Fe) and Ag/AgCl/MIL-101(Fe).

**Figure 3:**
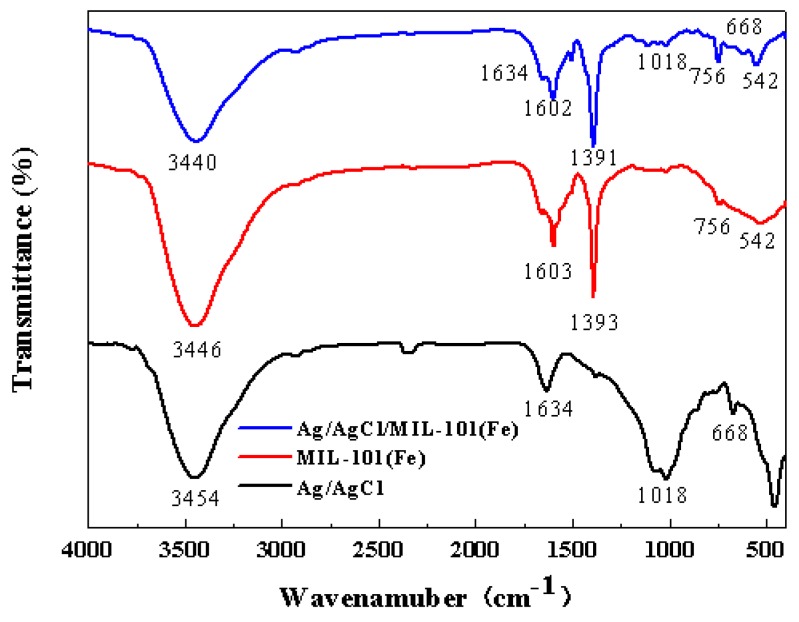
FTIR spectra of pure Ag/AgCl, MIL-101(Fe) and Ag/AgCl/MIL-101(Fe).

The nitrogen adsorption/desorption isotherms of MIL-101(Fe) and Ag/AgCl/MIL-101(Fe) are shown in Figure 2. Both of the samples exhibit a typical type IV pattern, indicating the existence of mesopores structure [30]. The horizontal and parallel adsorption/desorption branches suggest that these hysteresis loops are of type H4, reflecting the presence of narrow slit-shaped pores in the catalysts [31]. The BET surface area and pore volume of MIL-101(Fe) are 541.55 m^2^/g and 0.32 cm^3^/g, respectively. After deposited with Ag/AgCl, the corresponding values of Ag/AgCl/MIL-101(Fe) decrease to 355.86 m^2^/g and 0.31 cm^3^/g, which may be caused by the effect of pore blocking from the Ag/AgCl particles. To further analyze the molecular structure of the catalysts, the FTIR spectra of MIL-101(Fe) and Ag/AgCl/MIL-101(Fe) were performed. Also, the spectrum of Ag/AgCl was recorded for comparison, as shown in Figure 3. The Ag/AgCl presents the water OH stretching and bending vibration bands centered at around 3454 cm^−1^ and 1634 cm^−1^, respectively. Moreover, an absorption peak appears at around 668 cm^−1^, which corresponds to the typical stretching vibration of Ag–Cl bond [32]. For the MIL-101(Fe) sample, the sharp peak located at 1603 cm^−1^ and 1393 cm^−1^ are assigned to the asymmetric and symmetric stretching vibrations of O-C-O in carboxyl groups. The band at 756 cm^−1^ is characteristic of benzene rings, attributing to the C-H bending vibration. The band at 542 cm^−1^ is ascribed to the stretching vibration of Fe–O band in MIL-101(Fe) framework [33]. In the Ag/AgCl/ MIL-101(Fe), the absorption peaks at 1602, 1393, 756 and 542 cm^−1^ are the characteristic absorptions of MIL-101(Fe), while the peak around 668 cm^−1^ is the typical AgCl absorption peak, which is evidence the presence of AgCl on the surface of the MIL-101(Fe).

**Figure 4:**
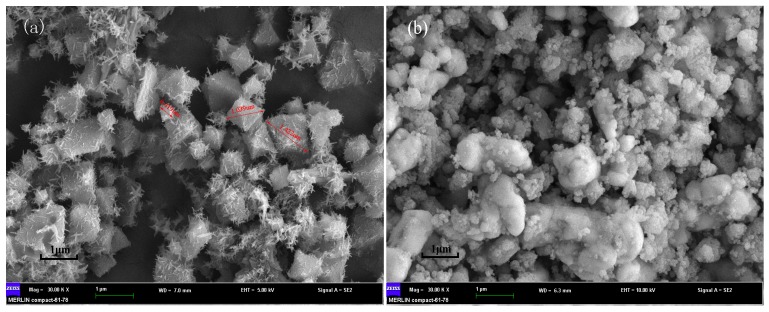
SEM images of MIL-101(Fe) (**a**) and Ag/AgCl/MIL-101(Fe) (**b**).

**Figure 5:**
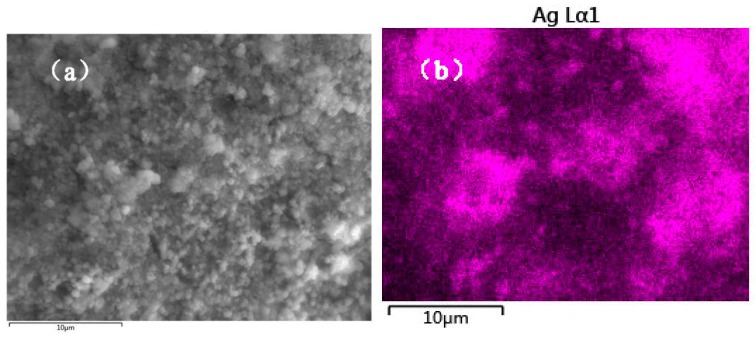

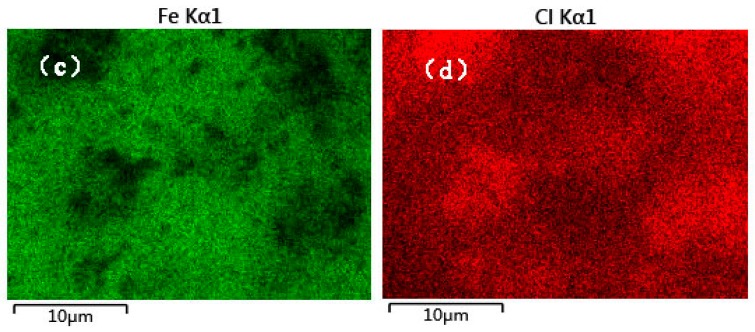
SEM image of Ag/AgCl/MIL-101(Fe) (**a**), SEM-EDX elemental mappings of Ag (**b**), Fe (**c**) and Cl (**d**).

The morphology of the MIL-101(Fe) and Ag/AgCl/MIL-101(Fe) were detected by SEM. As can be seen from Figure 4a,b, the as-prepared MIL-101(Fe) displays a typical octahedral structure with the sizes ranged from 0.4 to 1.4 μm. While the images of Ag/AgCl/MIL-101(Fe) reveal that clusters of Ag/AgCl were formed, resulting in a high coverage of the MIL-101(Fe). Furthermore, EDX elemental mapping for Ag/AgCl/MIL-101(Fe) (Figure 5) confirm the relative homogeneous distributions of Ag, Fe and Cl elements in the Ag/AgCl/MIL-101(Fe) sample.

**Figure 6:**
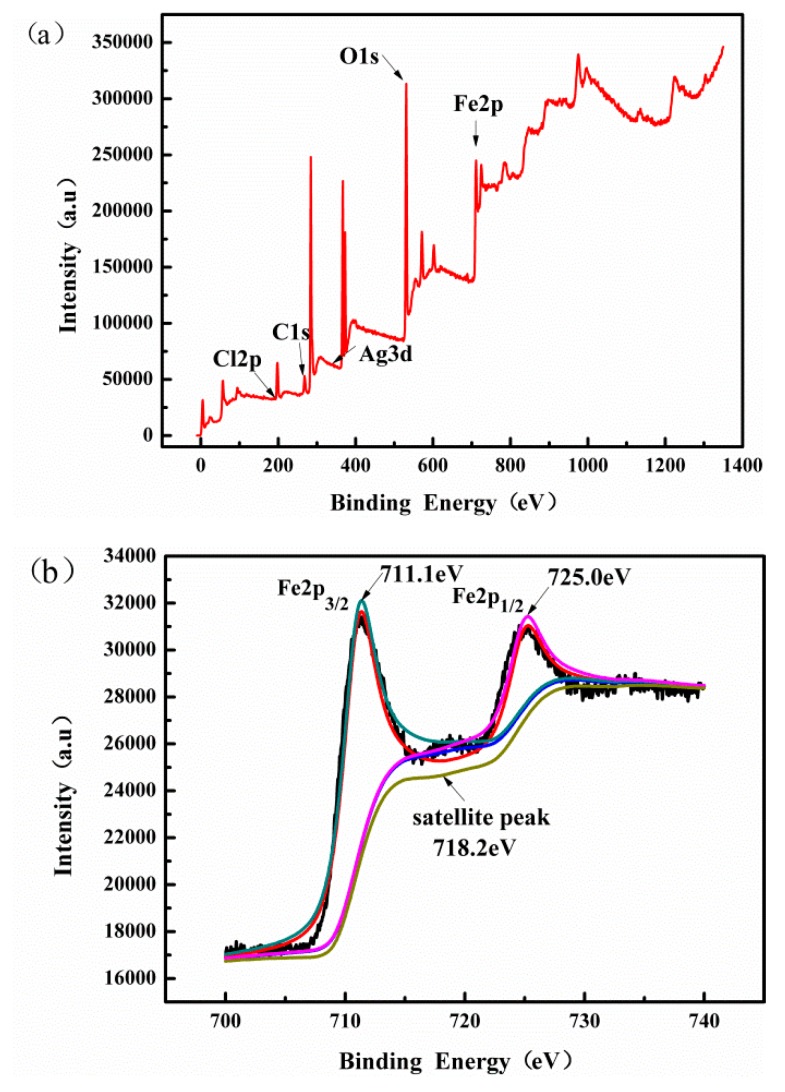

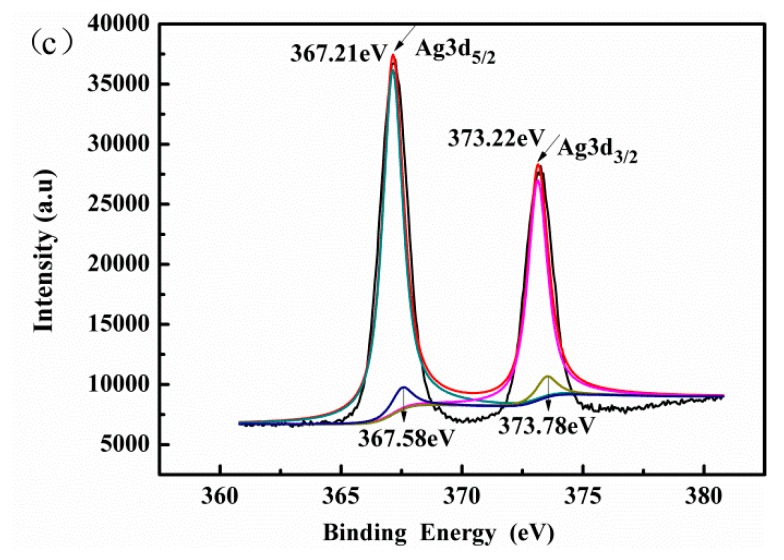
X-ray photoelectron survey spectrum (**a**), Fe 2p spectrum (**b**) and Ag 3d (**c**) spectrum of the Ag/AgCl/MIL-101(Fe).

The chemical composition of Ag/AgCl/MIL-101(Fe) was further investigated by XPS measurements (as shown in Figure 6). The XPS survey spectrum of the Ag/AgCl/MIL-101(Fe) sample (Figure 6a) shows the presence of Ag, Cl, and Fe elements, which agrees well with the above EDX result. The Fe 2p XPS spectrum of Ag/AgCl/MIL-101(Fe) (Figure 6b) indicates two well-defined peaks at 711.12 and 725.01 eV with a satellite signal at 718.21 eV, which are corresponding to Fe(Ⅲ) in MIL-101(Fe) [34]. In the Ag 3d XPS spectrum (Figure 6c), the peaks at 367.21 and 373.22 eV belong to Ag 3d 5/2 and Ag 3d 3/2 of Ag^+^, whereas the bands centered at 367.41 and 373.88 eV are attributed to metallic Ag^0^ according to previous reports [35]. The XPS measurements prove the existence of Ag/AgCl structure in Ag/AgCl/MIL-101(Fe) sample, which are agreeable to the XRD results.

**Figure 7:**
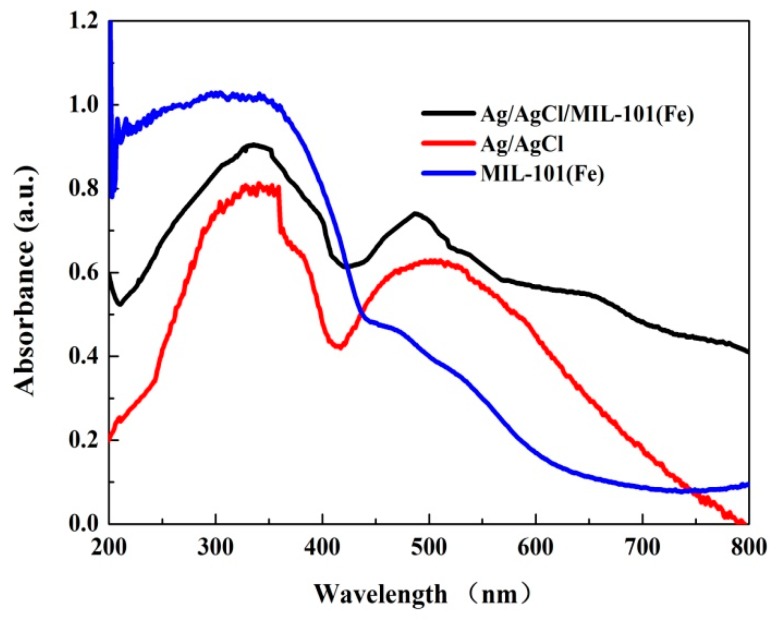
UV-vis diffuse reflectance spectra of the MIL-101(Fe) and Ag/AgCl/MIL-101(Fe).

The UV-vis diffuse reflectance spectra of Ag/AgCl, MIL-101(Fe) and Ag/AgCl/MIL-101(Fe) are compared in Figure 7. As can be seen, the MIL-101(Fe) shows only a strong photo-adsorption in the UV-light region. In contrast to the MIL-101(Fe), a remarkable enhancement of visible light absorption can be observed in the spectra of both Ag/AgCl and Ag/AgCl/MIL-101(Fe), which is ascribed to the surface plasmon resonance effect of Ag/AgCl [36]. The strong absorption in the visible light regions for the Ag/AgCl/MIL-101(Fe) implies its effective utilization of visible light energy for the photo-Fenton treatment of dyes.

### 3.2. Photo-Fenton Catalytic Activity

**Figure 8:**
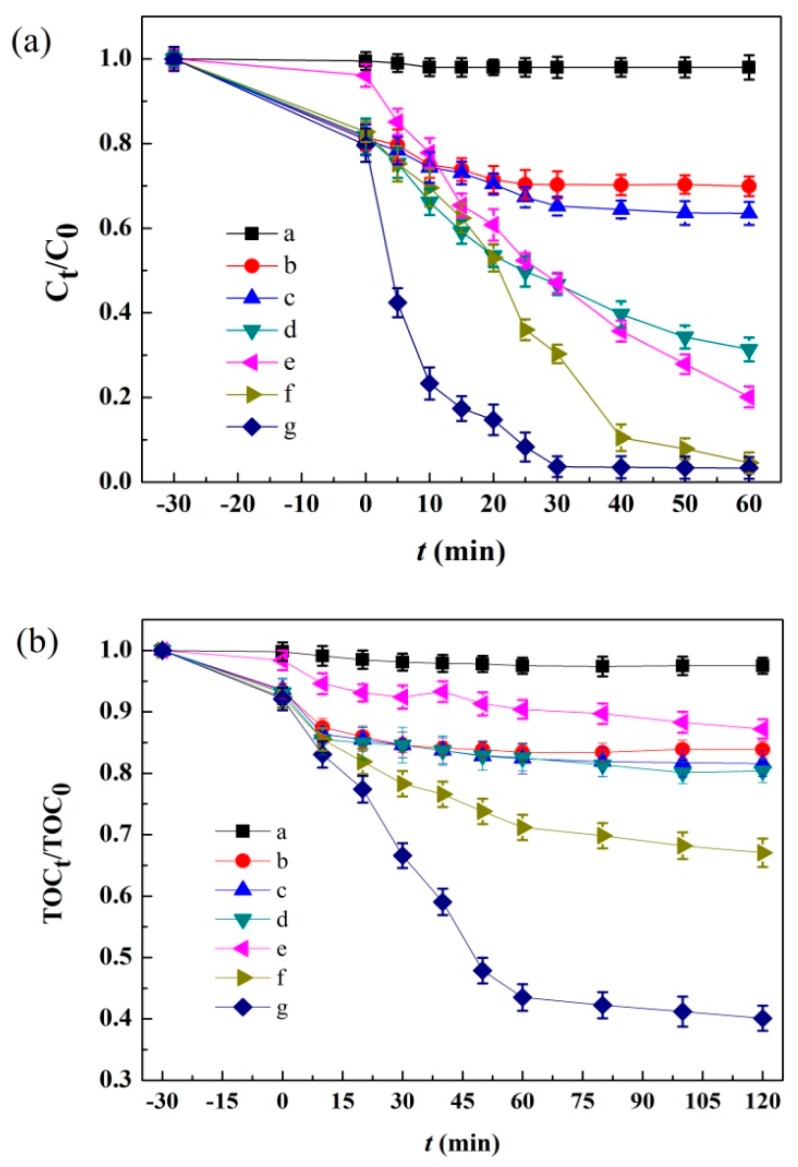
(**a**) Decoloration of MB under different conditions and (**b**) TOC removal of MB under different conditions. a.vis; b.Ag/AgCl/MIL-101(Fe); c.Ag/AgCl/MIL-101(Fe) + H_2_O_2_; d.vis + Ag/AgCl/MIL-101(Fe); e.vis + H_2_O_2_; f.vis + MIL-101(Fe) + H_2_O_2_; g.vis + Ag/AgCl/MIL-101(Fe) + H_2_O_2_; (pH = 7; [MB] = 10 mg/L; [H_2_O_2_] =10 mM; catalyst dosage = 1.0 g/L; radiation intensity = 500 W).

The photo-Fenton catalytic activity of the as-prepared Ag/AgCl/MIL-101(Fe) catalyst was studied using MB as the target contaminant under visible light irradiation, where control experiments were also carried out to compare the degradation efficiencies of MB in various conditions (as shown in Figure 8). It can be seen that MB is resistant to the oxidation under only visible light irradiation (curve a), as indicated by the 2.71% decolorization efficiency after 60 min and the only 1.42% of TOC removal efficiency after 120 min reaction. In the system with only Ag/AgCl/MIL-101(Fe) (curve b), approximately 30.12% of MB and 16.13% of TOC are removed, which is mainly ascribed to the adsorption of MB by the catalyst. In the presence of H_2_O_2_ and Ag/AgCl/MIL-101(Fe) (curve c), the decolorization and mineralization efficiencies of MB reach to 36.48% and 16.87% respectively, owing to the Fenton effect. For the system with visible light and Ag/AgCl/MIL-101(Fe) (curve d), the MB degradation efficiency of 68.65% and TOC removal efficiency of 18.36% are obtained, which are slightly higher than that in Ag/AgCl/MIL-101(Fe) system due to the photo catalytic effect of the catalyst. When the reactions are performed in the visible light + H_2_O_2_ system (curve e), the MB decolorization efficiency can increase to 79.87%. The high MB decolorization efficiency is attributed to the formation of OH· through the direct photolysis of H_2_O_2_ [37]. However, only 19.63% of TOC is removed in this system, implying that the OH radicals generated in this system are not enough to mineralize MB into H_2_O and CO_2_ [38]. In the system of MIL-101(Fe) + visible light + H_2_O_2_ (curve f) and Ag/AgCl/MIL-101(Fe) + visible light + H_2_O_2_ (curve g), significant decolorization efficiency of MB (more than 95%) can be observed. However, the Ag/AgCl/MIL-101(Fe) + visible light + H_2_O_2_ system shows much higher TOC removal efficiency (61.05%) and faster MB degradation rate, indicating that Ag/AgCl/MIL-101(Fe) can be used as an efficient heterogeneous photo-Fenton catalyst under visible light irradiation.

### 3.3. Fitting Model and Analysis of Variance (ANOVA)

The BBD experiments were conducted to study the effects of initial MB concentration, H_2_O_2_ concentration, catalytic dose and irradiation intensity on the MB degradation. Based on BBD experiment results, the relationship between the responses (MB decolorization efficiency and TOC removal efficiency) and variables were expressed as follows (Equations (4) and (5)):

(4) $$\begin{array}{l} \mathrm{MB}\;\mathrm{decolorization}\;\mathrm{efficiency}\\ \;=78.33-16.28A+7.45B+15.17C+16.52D-3.97\mathrm{AB}-1.53\mathrm{AC}-6.89\mathrm{AD}-7.38\mathrm{BC}\\ \;+5.86\mathrm{BD}-0.42\mathrm{CD}-12.39A^{2}-5.89B^{2}-13.39C^{2}-10.10D^{2} \end{array}$$

(5) $$\begin{array}{l} \mathrm{TOC}\;\mathrm{removal}\;\mathrm{efficiency}\\ \;=43.27-11.76A+4.79B+7.08C+10.87D-6.01\mathrm{AB}-2.15\mathrm{AC}-5.12\mathrm{AD}-1.11\mathrm{BC}\\ \;+4.18\mathrm{BD}-4.40\mathrm{CD}-6.50A^{2}-1.46B^{2}-6.69C^{2}-3.76D^{2} \end{array}$$

Here, A, B, C and D represent the initial concentration of dye, the catalytic dose, the concentration of hydrogen peroxide and the radiation intensity respectively.

The statistical models were analyzed by F-test, and the ANOVA results of the quadratic models are presented in Table 2. The correlation coefficient (R^2^) of 0.9821 and adjusted determination coefficient R^2^_adj_ of 0.9641 for MB decolorization efficiency and the R^2^ of 0.9737 and R^2^_adj_ of 0.9423 for mineralization efficiency are closer to 1, indicating that these regression models fit well with the experimental values and they can offer adequate explanations about the relationships between the responses and the independent variables [39]. The Model F-value of 54.76 and 13.23 for MB and TOC removal efficiency respectively suggest that these models are significant. Moreover, the models’ adequate precisions of 16.92 and 13.62 for decolorization efficiency and mineralization efficiency are much greater than four, implying acceptable signals for the models to be applied [40]. The model terms with the value of “probability > F” less than 0.0500 are significant [41]. In the MB removal efficiency case, the model terms of A, B, C, D, BD, CD, A^2^, B^2^, C^2^ and D^2^ are significant, while A, B, C, D, AB, A^2^ and C^2^ are significant model terms in the TOC removal case.

### 3.4. Response Surface Analysis

**Figure 9:**
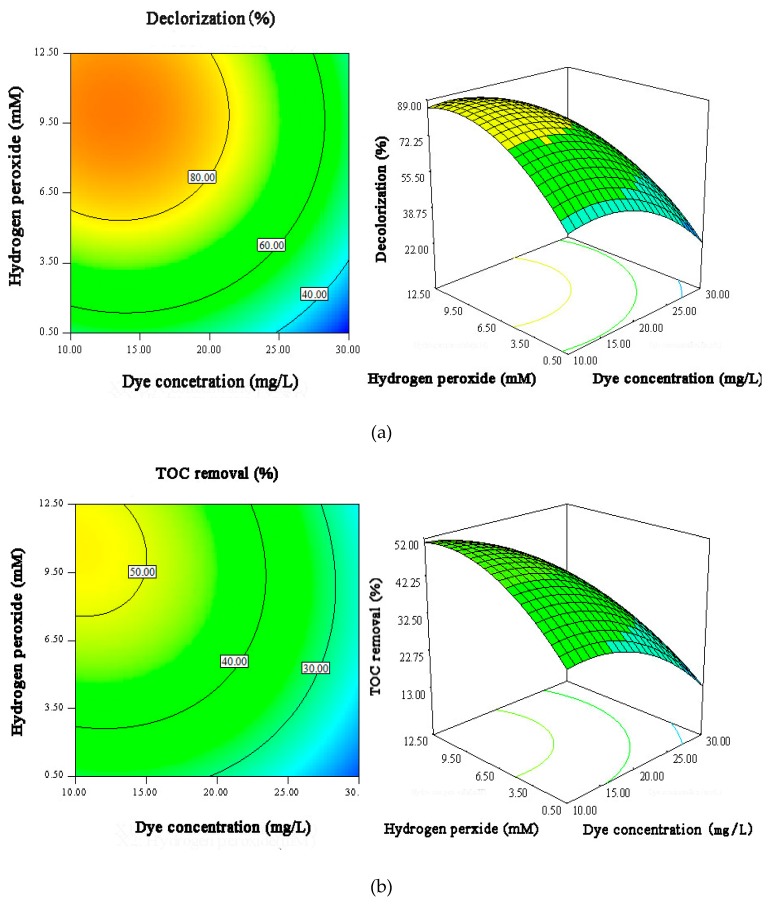
The contour plots and 3D surface responses for the effects of initial H_2_O_2_ concentration and MB concentration on MB decolorization efficiency (**a**) and TOC removal efficiency (**b**).

**Figure 10:**
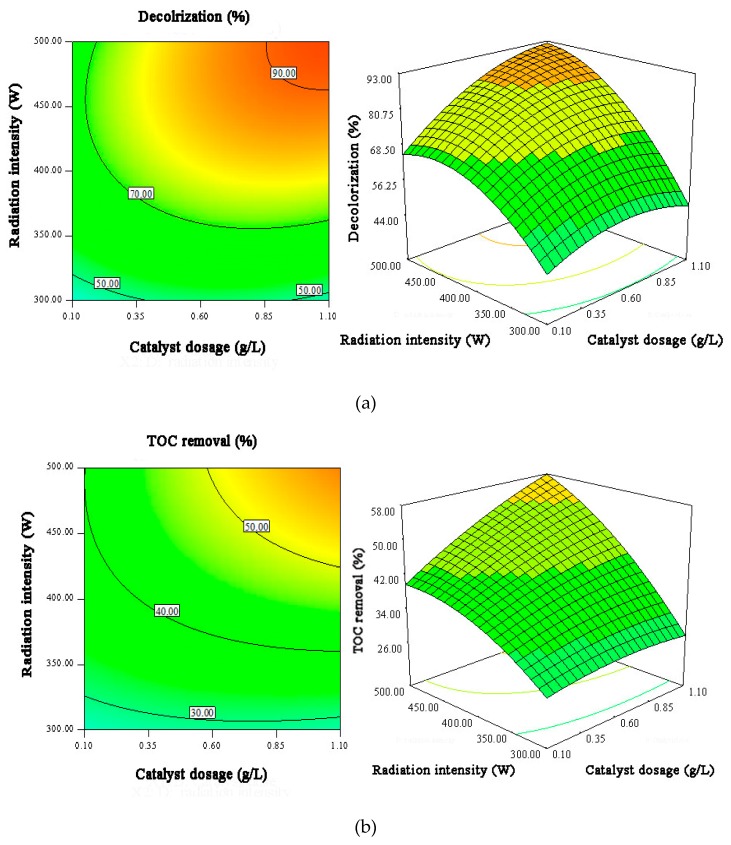
The contour plots and 3D surface responses for the effects of catalyst dosage and radiation intensity on MB decolorization efficiency (**a**) and TOC removal efficiency (**b**).

**Figure 11:**
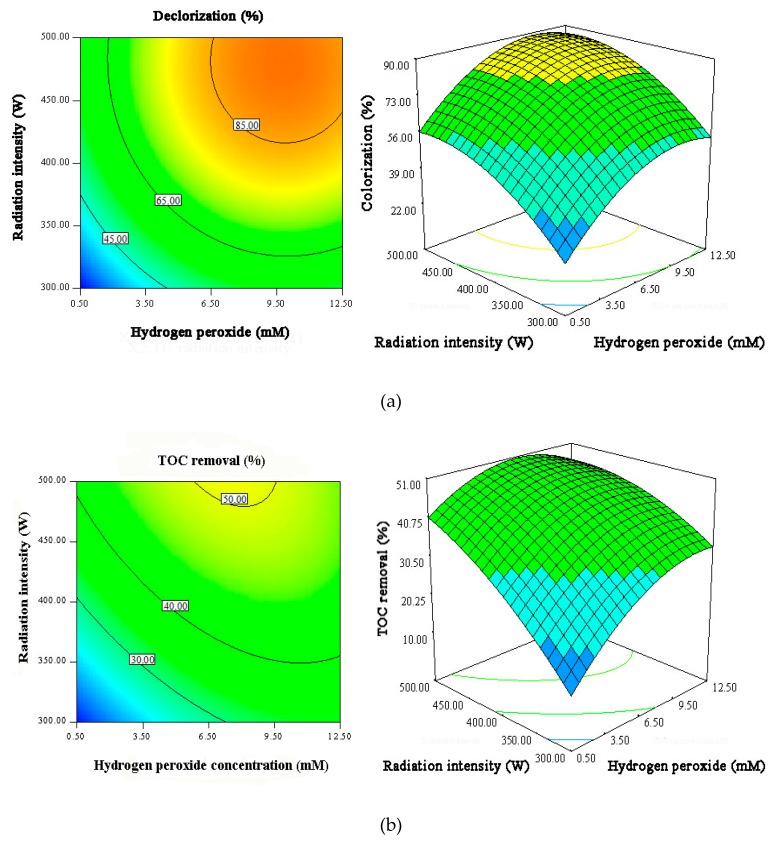
The contour plots and 3D surface responses for the effects of initial H_2_O_2_ concentration and radiation intensity on MB decolorization efficiency (**a**) and TOC removal efficiency (**b**).

The response surface results shown in Figure 9, Figure 10 and Figure 11 directly describe the effects of the main variables of Ag/AgCl/MIL-101(Fe) photo-Fenton catalytic degradation process on the MB and TOC removal. 

The interactions of initial H_2_O_2_ concentration and MB concentration on the MB decolorization and mineralization efficiencies were shown in Figure 9. Both of MB decolorization efficiency and TOC removal efficiency increase with the increase of H_2_O_2_ at low concentration, due to the production of more OH· or other free radicals by higher concentration of H_2_O_2_ in a certain range. However, further increase of H_2_O_2_ will lead to the scavenging of OH· radicals, resulting in a slight decrease of MB degradation efficiency [42]. Additionally, Figure 9 also demonstrates that the MB decolorization and mineralization efficiencies decrease with increasing of initial MB concentration, which is result from the fact that the higher dye concentrations can affect the permeability of the solution and thus result in a decrease of the visible light utilization efficiency [43].

Figure 10 shows the interactions of irradiation intensity and catalyst load on the MB decolorization efficiency and TOC removal efficiency. It can be seen that radiation intensity has a more notable impact than catalyst dosage on MB degradation efficiency. As the irradiation intensity increases from 300 to 500 W, the MB decolorization and mineralization efficiencies increase significantly. The reason is that more photons are generated at higher intensity of irradiation, which can effectively accelerate the photo-Fenton reaction [44]. Also, the MB degradation efficiency is enhanced by increasing the catalyst dosage in the studied catalyst concentration range, owing to the increase in the accessible active sites of the catalyst [45]. However, excessive catalyst in solution may result in decreased visible light penetration owing to the screening effects.

It also can be seen that the influence of catalyst dosage on the MB degradation is much significant in the system with higher light irradiation. The irradiation intensity has a notable influence in the reaction owing to its influence on the photon’s generation. When lower irradiation intensity is applied, limited photons are produced in the solution, which results in the less activation of Ag/AgCl/MIL-101(Fe) catalyst. This phenomenon leads to the less significant influence of catalyst dosage on the MB degradation at lower irradiation intensities. When sufficient photons are generated in the system at the higher light intensity, the catalytic active sites become much more important. Thus, the influence of the catalyst dosage on the MB degradation becomes much more significant.

The effects of initial H_2_O_2_ concentration and irradiation intensity on the MB degradation (Figure 11) reveal that 90% of MB removal efficiency and 50% of TOC removal efficiency can be realized at irradiation intensity higher than 450 W and H_2_O_2_ concentration higher than 9.5 mmol. Of course, the interrelation between the variables is important for the optimization of MB degradation. 

### 3.5. Model Validation and Experimental Confirmation

Based on the results of BBD, the optimal process parameters are determined to be 10.72 mg/L of MB concentration, 0.97 g/L of catalyst dosage, 10.90 mM of H_2_O_2_ concentration and 457.79 W of radiation intensity with the maximum MB decolorization efficiency of 100% and maximum TOC removal of 65.63%. To confirm the reliability of the predicted models, three additional experiments under these optimal conditions were conducted. The average MB decolorization and mineralization efficiencies are 99.75% and 65.43% respectively, confirming that the BBD was effective and reliable in the photo-Fenton optimization experiments.

### 3.6. Recyclability

**Figure 12:**
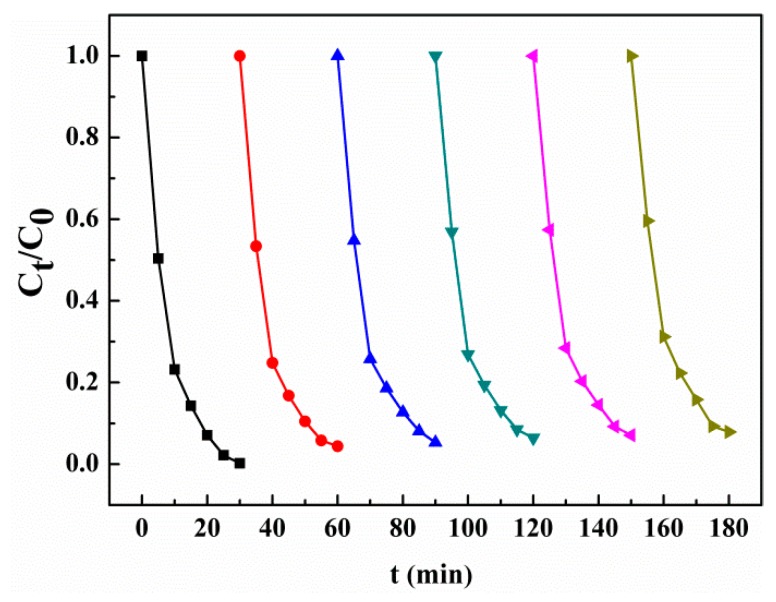
The cycling runs of MB degradation in the photo-Fenton system catalyzed with Ag/AgCl/MIL-101(Fe).

The stability of Ag/AgCl/MIL-101(Fe) in the photo-Fenton reaction was studied by means of recycling experiments under the above-mentioned optimum conditions. As shown in Figure 12, an MB decolorization efficiency above 99.8% is achieved in the first run of reaction. After six repeated reactions, the MB decolorization efficiency can still be kept at 92.1%. 

**Figure 13:**
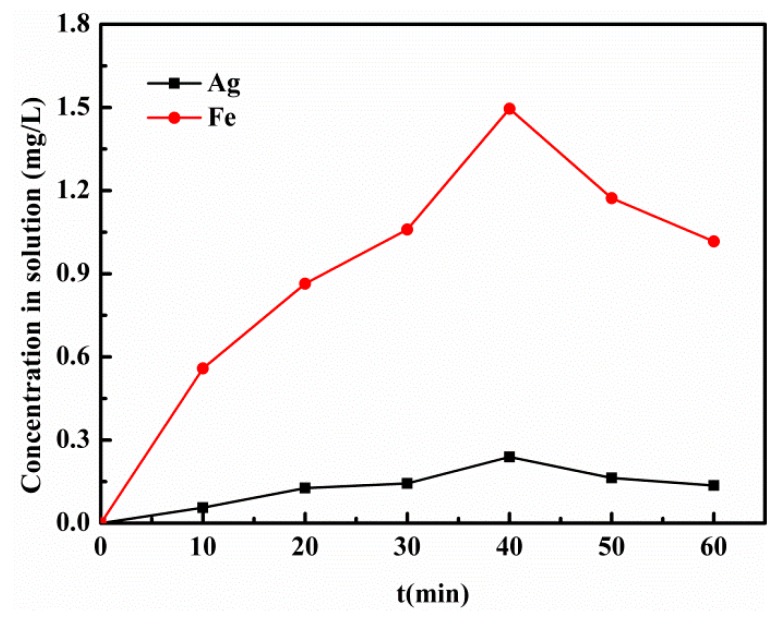
The concentrations of Ag and total Fe ions in solution as a function of time in the photo-Fenton process.

Additionally, the concentrations of Ag and total Fe ions in solution as a function of time in the photo-Fenton process were measured by atomic absorption spectrophotometer (Figure 13), which shows that the highest concentration of Ag and total Fe ions is about 0.17mg/L and 1.58mg/L, respectively. Compared to the content of Ag and Fe in the catalyst, the leached Ag and Fe ions in the studied system are relatively low (about 0.76% and 1.81%, respectively). Hence, the results show that the Ag/AgCl/MIL-101(Fe) catalyst exhibits good reusability and stability under visible light irradiation. 

Besides, it should be noted that the purpose of this study is to test the activity of the synthesized catalyst and explore its photo-Fenton catalytic mechanism. The real wastewater is too complex to achieve this purpose. Therefore, the experiment was carried out under laboratory conditions but not in an actual wastewater system. Of course, the real wastewater treatment will be carried out in the future studies so as to test the practical value of the catalyst.

### 3.7. Proposed Photo-Fenton Mechanism

**Figure 14:**
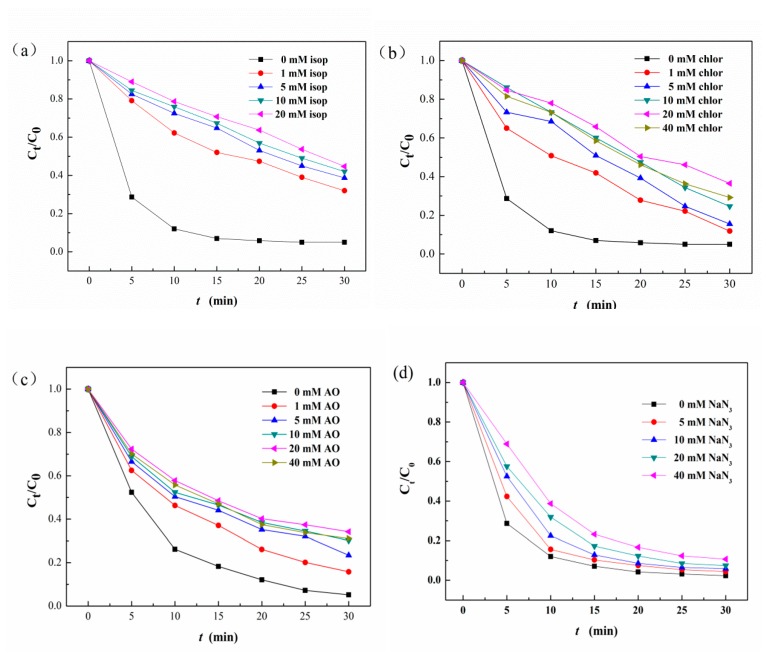
The influence of ammonium oxalate (**a**), chloroform (**b**) isopropyl alcohol (**c**) and sodium azide (**d**) on the MB decolorization efficiency. (pH = 7; [MB] = 10 mg/L; [H_2_O_2_] = 10 mM; catalyst dosage = 1.0 g/L; radiation intensity = 500 W).

To investigate the reactive species of the photo-Fenton process using Ag/AgCl/MIL-101(Fe) as catalyst for MB degradation, radical capture experiments were carried out by using a series of radical scavengers. In this study, isopropyl alcohol (IPA), chloroform, ammonium oxalate (AO) and sodium azide (NaN_3_) were adopted as the scavengers for hydroxyl radical (·OH), superoxide radical (O_2_·^−^), holes (h^+^) and singlet oxygen (^1^O_2_), respectively. As shown in Figure 14a–c, the maximum inhibition of MB degradation is up to 50.80%, 31.50 % and 28.60% after 30 min reaction when 20 mM IPA, 20 mM chloroform, and 20 mM AO is added in the solution, respectively. These results suggest that ·OH plays the most important role in the degradation of MB, followed by O_2_·^−^ and h^+^. However, the addition of NaN_3_ presents relatively slight effect on MB degradation as shown in Figure 14d, indicating ^1^O_2_ is not the main reactive specie involved in the degradation of MB.

**Figure 15:**
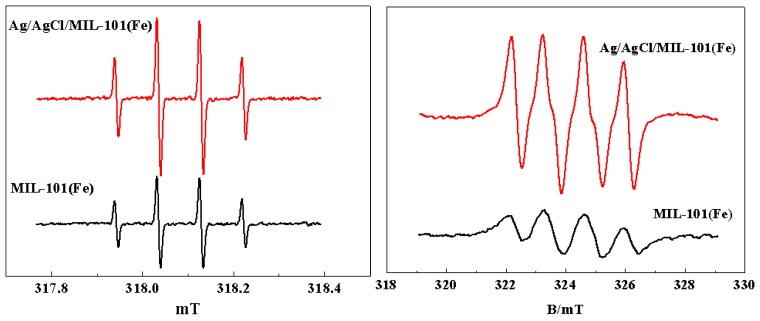
The ESR spectra of DMPO-HO· adducts (**a**) and DMPO-O_2_·^−^ adducts (**b**) in the photo-Fenton system with MIL-101(Fe) and Ag/AgCl/MIL-101(Fe).

ESR spin trapping experiment using DMPO as a spin trap were also carried out to confirm the generation of ·OH and O_2_·^−^ in the photo-Fenton system with Ag/AgCl/MIL-101(Fe). For comparison, the ESR spectra measured in the photo-Fenton system with MIL-101(Fe) were also given, which are shown in Figure 15. As can be seen, the four-fold characteristic peak corresponding to DMPO–OH· adduct with a peak intensity ratio of 1:2:2:1 can be clearly observed in the photo-Fenton system catalyzed with Ag/AgCl/MIL-101(Fe), while the peak strength under the photo-Fenton system with MIL-101(Fe) is relatively weaker. Similarly, the signal intensity of DMPO-O_2_^−^ adduct in the photo-Fenton system with Ag/AgCl/MIL-101(Fe) is much higher than that of the system with MIL-101(Fe). This fact can be used to explain why Ag/AgCl/MIL-101(Fe) has much higher catalytic activity than MIL-101(Fe).

**Figure 16:**
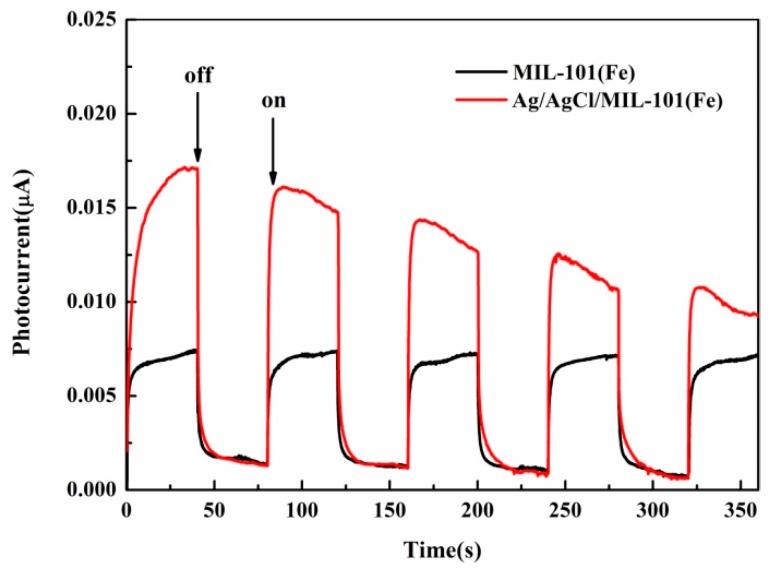
Transient photocurrent responses of Ag/AgCl/MIL-101(Fe) and MIL-101(Fe).

Additionally, the photocurrent responses of Ag/AgCl/MIL-101(Fe) and MIL-101(Fe) were analyzed and the results are illustrated in Figure 16. As is shown, both of the materials can generate photocurrent with a reproducible response to on/off cycles of visible light irradiation. However, Ag/AgCl/MIL-101(Fe) presents much higher photocurrent density than that of pure MIL-101(Fe), indicating the higher light harvesting and more effective of photo-induced electron transfer for Ag/AgCl/MIL-101(Fe). This result illustrates that the modification of Ag/AgCl can greatly enhanced the visible light activity of MIL-101(Fe).

**Figure 17:**
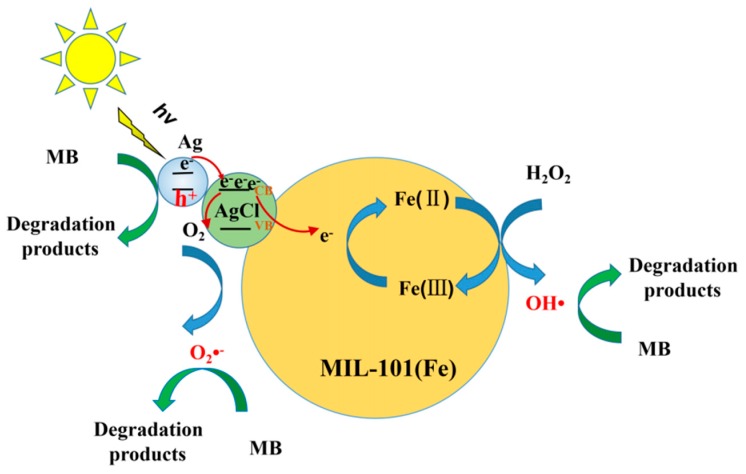
The proposed mechanism for the photo-Fenton degradation of MB using Ag/AgCl/MIL-101(Fe) as catalyst.

According to the above results, the mechanism MB degradation in the of heterogeneous photo-Fenton system using Ag/AgCl/MIL-101(Fe) as catalyst was proposed (shown in Figure 17). Under visible light irradiation, the Ag nanoparticles on the surface of Ag/AgCl/MIL-101(Fe) catalyst adsorb the visible light photons and generate a large amount of electron and hole pairs due to the surface plasmon resonance effect [46]. The photo-induced electrons rapidly migrate to the conduction band (CB) of AgCl, and the leaving holes will react with the MB in solution to produce the corresponding degradation products. Also, on one hand, the electrons in CB of Ag/AgCl are captured by Fe(III) on the surface of MIL-101(Fe) to generate the Fe(II), which can promote Fe(III)/Fe(II) cycle and accelerate the Fenton reaction. This is the reason why OH· radical is the main reactive specie in the system. On another hand, the electrons can be absorbed by dissolved O_2_ molecules so as to produce O_2_·^−^ radical, which also plays an important role in the degradation of MB.

## 4. Conclusions

In this paper, a novel material Ag/AgCl/MIL-101(Fe) was successfully synthesized and used as heterogeneous photo-Fenton catalyst for the treatment of MB at near-neutral pH under visible light irradiation. PBD and BBD methods were employed to screen and optimized the variables which influenced the degradation of MB, and the optimal conditions were obtained. The results showed that Ag/AgCl/MIL-101(Fe) exhibited high catalytic activity in the visible light-Fenton system. Under the optimal conditions, 99.75% of MB decolorization efficiency and 65.43% of TOC removal efficiency was achieved. The recycling experiments also indicated that the as-prepared catalyst had excellent stability. In addition, the reactive species trapping experiments revealed that OH·, O_2_·^−^ and h^+^ exhibited synergistic effects in the photo-Fenton system using Ag/AgCl/MIL-101(Fe) as catalysts for MB degradation.

## Figures and Tables

**Table 1:** Experimental range and levels of the independent variables.

Influence Factors	Code	Levels Low (−)	Levels Middle (0)	Levels High (+)
Dye concentration(mg/L)	A	10	20	30
Hydrogen peroxide(mM)	B	0.5	6.5	12.5
Catalyst dosage(g/L)	C	0.2	0.7	1.2
Radiation intensity (W)	D	300	400	500

**Table 2:** ANOVA results of the quadratic models for MB degradation.

Source		Decolorization				TOC Removal			
	D	Sum of	Mean	F Value	Prob > F	Sum of	Mean	F Value	Prob > F
	F	Square	Square			Square	Square		
Model	14	12376.46	12376.46	54.76	<0.0001	4873.04	4873.04	13.23	<0.0001
A	1	3178.48	3178.48	133.59	<0.0001	1658.0	1658.0	63.06	<0.0001
B	1	665.88	665.88	128.72	0.0017	274.85	274.85	10.45	0.006
C	1	2761.85	2761.85	164.48	<0.0001	601.83	601.83	22.88	0.0003
D	1	3274.59	3274.59	211.95	<0.0001	1418.32	1418.32	53.92	<0.0001
AB	1	63.04	63.04	12.21	0.2547	144.36	144.36	5.49	0.0344
AC	1	9.33	9.33	0.074	0.6542	18.53	18.53	0.70	0.4154
AD	1	190.16	190.16	54.13	0.0528	104.96	104.96	3.99	0.0656
BC	1	22.71	22.71	36.71	0.1017	4.96	4.96	0.19	0.6224
BD	1	137.1	137.1	36.94	0.0001	69.81	69.81	2.65	0.1256
CD	1	0.71	0.71	0.0052	0.0054	77.44	77.44	2.94	0.1082
A^2^	1	996.37	996.37	22.29	0.0003	274.14	274.14	10.42	0.0061
B^2^	1	225.13	225.13	5.04	0.0415	13.80	13.80	0.52	0.4808
C^2^	1	1162.34	1162.34	26.01	0.0002	290.51	290.51	11.04	0.0050
D^2^	1	661.86	661.86	14.81	0.0018	91.69	91.69	3.49	0.0830
Residual	14	625.67	44.69			368.28	26.31		
Pure Error	4	0	8.330 × 10^−3^			0	1.770 × 10^−3^		
R^2^ = 0.9821; R^2^_adj_ = 0.9641						R^2^ = 0.9737; R^2^_adj_ = 0.9423			
R^2^_pred_ = 0.9021; adeq precision = 16.920						R^2^_pred_ = 0.8577; adeq precision = 13.62			

## Supplementary Materials

The following are available online at https://www.mdpi.com/1996-1944/12/9/1453/s1, Table S1: Factors and levels in Plackett-Burman design. Table S2: Effects of the variables of the Plackett-Burman design.
