# Supplementary material for: Ag/AgCl/MIL-101(Fe) Catalyzed Degradation of Methylene Blue under Visible Light Irradation
Source: Materials (Basel). 2019 May 5;12(9):1453. doi: 10.3390/ma12091453 (PMC6540000; doi:10.3390/ma12091453)
Supplement: Supplementary file 1 [file materials-12-01453-s001.pdf]

materials

Supplementary Materials

Ag/AgCl/MIL-101(Fe) Catalyzed Degradation of Methylene Blue under Visible Light Irradation

Yun Liu [1,*], Yuanhong Xie [1], Mingjin Dai [1], Qingjiao Gong [1] and Zhi Dang [2]

[1] Department of Environmental Science and Engineering, College of Environment and Resources, Xiangtan University, Xiangtan 411105, China; xieyuanhong0222@126.com (Y.X.); dmj19930611@163.com (M.D.); qingjiaogong@163.com (Q.G.)
[2] School of Environment and Energy, South China University of Technology, Guangzhou 510006, China; chzdang@scut.edu.cn
* Correspondence: liuyunscut@163.com; Tel.: +86-181-7327-7750

Table S1. Factors and levels in Plackett-Burman design.

Factor	Name	Units	Levels low (–)	Levels high (+)
X1	Initial pH	—	5	9
X2	Initial dye concentration	mg/L	10	30
X3	catalyst dosage	g/L	0.1	1.1
X4	Initial hydrogen peroxide	mM	0.5	12.5
X5	radiation intensity	W	300	500
X6	adsorption time	min	10	60
X7–X11	virtual variables	—	—	—

Table S2. Effects of the variables of the Plackett-Burman design.

Term	Stdized Effects	Sum of Squares	Contribution (%)
Initial pH	-1.44	6.19	0.17
initial MB concentration	-19.66	1157.19	31.73
catalyst dosage	14.18	602.93	16.53
initial H_2O_2 concentration	15.71	740.10	20.29
irradiation intensity	18.72	1050.94	28.81
adsorption time	2.21	14.70	0.40

© 2019 by the authors. Licensee MDPI, Basel, Switzerland. This article is an open access article distributed under the terms and conditions of the Creative Commons Attribution (CC BY) license (http://creativecommons.org/licenses/by/4.0/).
